# The Music of Your Emotions: Neural Substrates Involved in Detection of Emotional Correspondence between Auditory and Visual Music Actions

**DOI:** 10.1371/journal.pone.0019165

**Published:** 2011-04-29

**Authors:** Karin Petrini, Frances Crabbe, Carol Sheridan, Frank E. Pollick

**Affiliations:** Department of Psychology, University of Glasgow, Glasgow, Scotland; McGill University, Canada

## Abstract

In humans, emotions from music serve important communicative roles. Despite a growing interest in the neural basis of music perception, action and emotion, the majority of previous studies in this area have focused on the *auditory* aspects of music performances. Here we investigate how the brain processes the emotions elicited by *audiovisual* music performances. We used event-related functional magnetic resonance imaging, and in Experiment 1 we defined the areas responding to audiovisual (musician's movements with music), visual (musician's movements only), and auditory emotional (music only) displays. Subsequently a region of interest analysis was performed to examine if any of the areas detected in Experiment 1 showed greater activation for emotionally mismatching performances (combining the musician's movements with mismatching emotional sound) than for emotionally matching music performances (combining the musician's movements with matching emotional sound) as presented in Experiment 2 to the same participants. The insula and the left thalamus were found to respond consistently to visual, auditory and audiovisual emotional information and to have increased activation for emotionally mismatching displays in comparison with emotionally matching displays. In contrast, the right thalamus was found to respond to audiovisual emotional displays and to have similar activation for emotionally matching and mismatching displays. These results suggest that the insula and left thalamus have an active role in detecting emotional correspondence between auditory and visual information during music performances, whereas the right thalamus has a different role.

## Introduction

In everyday life, we continuously have to make up our minds how we feel about certain ‘things’ or ‘people’, in order to know how to behave appropriately. To achieve a coherent representation of our subjective feelings about external situations, our brain needs to process and integrate the emotional information coming from different sensory modalities. When the emotional state communicated by sight and sound is the same (e.g. a person speaking with a positive tone of voice and acting in a positive way) the task may be effortless, however, in everyday life we are also faced with situations where the emotional state communicated by one sensory modality is different from the emotional state communicated by the other (e.g. a person speaking with a positive tone of voice but acting in a negative way). Hence, our brain should be able to process and resolve this mismatching emotional information.

In humans, emotions from music serve important communicative roles, and as observers we have the capacity to appreciate the emotional content of musicians' performances from their audiovisual output. Although the biological value of music is far from being understood [1], familiar as well as unfamiliar music is able to induce strong emotions with positive and negative valences [2]–[4], which is why music has become an increasingly popular tool for studying emotional processes in the brain. In an elegant PET study, Blood et al. [1] used pleasant (e.g. consonant) and unpleasant (e.g. dissonant) melodies to investigate the underlying emotional processes, and showed that activation of the right parahippocampal gyrus and the precuneus correlated with an increase in unpleasantness, whilst activation of the frontopolar, orbitopolar, and subcallosal cingulate cortex correlated with a decrease in unpleasantness. In a subsequent PET study, Blood and Zatorre [5] confirmed and extended their previous findings, by showing that an increase of an intensely pleasurable response to music (in the form of “chills”) correlated with activity in the left ventral striatum, right dorsomedial midbrain, right thalamus, left anterior cingulate, right orbifrontal cortex, bilateral insula, right supplementary motor area, and bilaterally in the cerebellum. In contrast, a decrease in pleasurable response was found to correlate with activity in the left hippocampus/amygdala, right amygdala, bilaterally in the medial prefrontal cortex, right cuneus, and bilaterally in the precuneus. These findings indicate that several areas are involved in the perception of emotions from music, and that only a subset of these areas are activated depending on the emotional valence of the stimulus. For example, Blood and Zatorre [5] pointed to the lack of correlation between activity in the parahippocampal gyrus and perceived chills as demonstration of the specific participation of this area in the processing of negative emotions.

Recently, an fMRI study by Koelsch et al. [6], with a similar approach to those of Blood and colleagues, used naturally pleasant and unpleasant musical excerpts, instead of computerised excerpts, to investigate emotion. The authors contrasted the brain activity for pleasant music with the activity for unpleasant music and showed that, when both experimental blocks where considered, Heschl's gyrus, the left inferior frontal gyrus (BA45/46), and the anterior superior insula were active, whilst the opposite contrast showed activation in the hippocampus, parahippocampal gyrus, and temporal pole. Despite some small dissimilarities, all of these studies found a number of consistent results. For example, the activity in the parahippocampal gyrus and amygdala was found in response to the modulation of primarily negative emotion elicited by music. On the other side, the ventral striatum was primarily found to respond to positive emotion elicited by music. However, it is still somewhat unclear whether any of these brain regions are also responsible for processing the multisensory emotional information induced by the sound and sight of musical actions, or whether a completely separate network of brain regions is responsible for the integration of the emotional information coming from different sensory modalities.

Some information on which areas may participate in the processing of multisensory emotional information is brought to us by recent studies using face/voice emotional stimuli [7], [8]. These studies consistently indicate that the STG and the right thalamus participate in the integration of audiovisual emotional signals from speech, in that these regions were showing increased activation for multimodal emotional stimuli when compared to unimodal, and their activation correlated with the measure of multimodal facilitation in correctly judging the perceived emotion [8]. Since no brain imaging study has yet addressed this aspect for music, in the present study we aim to examine which brain regions are responsible for processing audiovisual emotional signals and detecting audiovisual emotional correspondence from music displays. Because right thalamus activity was found to increase in response to an increase in chills from music as well as in response to an increase in multimodal facilitation when judging emotional multisensory speech stimuli, we may assume that this area is a probable candidate in integrating audiovisual emotional signals from music. On the other side, the insula has been found to have a primary role in processing the emotional aspects of music [6], [9]–[12]. Its role is not limited to auditory information but extends to cases where emotional music pieces are combined with emotionally congruent pictures [13]. The suggestion that the insula is involved in the detection of crossmodal correspondence [14], [15] makes the insula the most probable candidate for detecting emotional correspondence between the sight and sound of music actions.

To address this point, we first carried out an experiment by using music improvisation performances expressing sadness, happiness and surprise [4] to examine which brain regions respond to musical displays presenting both emotional sight and sound, only sound and only sight. Subsequently, the brain regions detected in this first experiment were used as regions of interest (ROIs), as we examined which of these areas would show greater activation for mismatching (musician's movements with mismatching emotional music) than matching (musician's movements with matching emotional music) emotional displays.

## Methods

### Participants, Stimuli and Task

Sixteen right-handed participants (mean age 21.75 years, range 19–24 years: 8 females and 8 males) of UK nationality and with little or no experience with musical instruments participated in two functional magnetic resonance imaging (fMRI) experiments. All participants had normal or corrected to normal vision and normal hearing, and were in good health with no past history of psychiatric or neurological disease. All participants gave informed written consent to the protocol, which had been approved by the Ethics Committee of the Faculty of Information and Mathematical Sciences, University of Glasgow.

**Figure 1:**
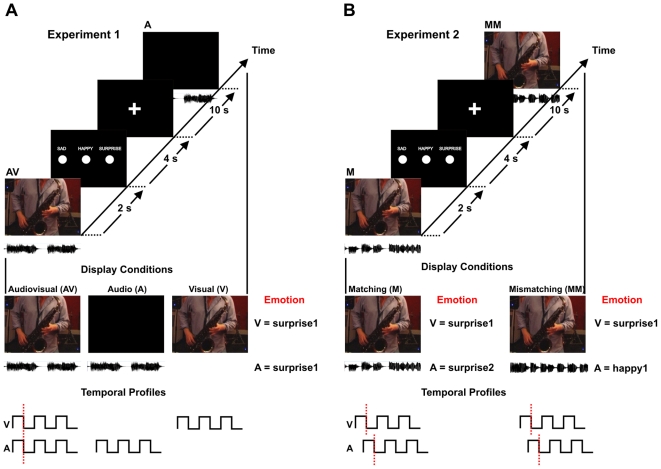
Schematic of stimulus conditions and experimental design in Experiments 1 and 2. (**A**) **Experiment 1.** Participants were scanned during three 7-minute runs during which the audiovisual (AV), visual-only (V) and auditory-only (A) stimuli were presented. In each run participants were shown 27 stimuli (3 emotions: sad, happy, and surprise X 3 displays X 3 modalities: audiovisual, auditory, and visual) for a total of 81 presentations. The audiovisual condition consisted of the original musical displays (2 s) in which auditory and visual signals were in synchrony, as illustrated in the temporal profiles at the bottom of the figure. The visual-only condition showed the visual displays extracted from the original audiovisual displays without any sound. The auditory displays were presented by showing a black screen together with the auditory signal. (**B**) **Experiment 2.** Participants were scanned during three 6-minute runs during which the emotionally matching (M) and mismatching (MM) displays were presented. In each run participants were shown 24 stimuli, of which 6 were emotionally matching and 18 were emotionally mismatching, for a total of 72 presentations. The emotionally matching displays combined visual and auditory signals that expressed the same emotion but were extracted from different original audiovisual displays. In contrast, the emotionally mismatching displays combined visual and auditory information that expressed different emotions and was extracted from different original audiovisual displays. In this way both types of emotionally matching and mismatching stimuli were presented as asynchronous visual and auditory signals, as illustrated in the temporal profiles at the bottom of the figure.

We scanned participants while they watched and listened to a saxophonist improvising three different emotions (sadness, happiness and surprise). We decided to use three emotional categories instead of two (which would have increased the statistical power and maintained a ratio 1∶1 when contrasting mismatching with matching displays), because we wanted to generalise our results to different kinds of emotions. Indeed, while happiness is a positive emotion and sadness is negative, surprise is somehow ambivalent as it can be perceived positively or negatively. The stimuli had previously been standardised by means of a series of behavioural experiments [4], and showed only the torso of the musician (Figure 1) in order to exclude the facial expressions. The visual stimuli consisted of 25 fps displays (PAL — 720×526) and were presented through fMRI compatible NNL (NordicNeuroLab) goggles so that binocular fusion could be optimised for each participant. The auditory stimuli were recorded and presented at 44100 Hz and had a duration of 2 s. The range of intensity levels (75–89 dB) at the sound source was the same for all stimuli and scanning sessions and was clearly audible above the scanner noise. Because the sound was clearly audible we decided against using less efficient sparse experimental designs that incorporate long periods of silence. The sound was presented to participants through fMRI compatible NNL headphones.

Musical improvisation was used instead of pre-existing pieces of music to make sure that participants shared the same level of familiarity with the music stimuli, and that the elicited emotional states in participants were due to the music displays and not to memories linked to familiar pieces of music. The basic event-related design was kept the same for both fMRI experiments (Figure 1). After each stimulus presentation (which lasted 2 s) participants were reminded of the possible responses (sad, happy, surprise) by presentation of a 4 s response screen followed by a 10 s ISI (Inter Stimulus Interval) which consisted of a black screen with a fixation cross at its centre. Participants were asked, while lying in the scanner, to choose the perceived emotion when the response screen was presented by pressing one of three buttons on an fMRI compatible button pad. The emotional classification task during the scan was kept the same in the two experiments to make sure that any difference between the results of the two experiments could not be explained by difference in behavioural tasks. To optimise the order of stimulus presentation within each run we used a 1-back genetic algorithm [16], which takes into consideration the number of stimuli for each condition, the length of ISI and the previously presented stimulus in order to create a sequence of stimulus presentation that optimises the BOLD (blood oxygen level dependent) signal change from baseline. In Experiment 1 participants were scanned during three 7-minute runs showing audiovisual (AV), visual-only (V) and auditory-only (A) stimuli. In each run participants were shown 27 stimuli (3 emotions: sad, happy, and surprise X 3 displays X 3 modalities: audiovisual, auditory, and visual) for a total of 81 presentations. The audiovisual condition consisted of the original musical displays (2 s) in which auditory and visual signals were in synchrony, as illustrated in the temporal profiles at the bottom of Figure 1 (left). The visual-only condition showed the visual displays extracted from the original audiovisual displays without any sound. The auditory displays were presented by showing a black screen together with the auditory signal. In Experiment 2 participants were scanned during three 6-minute runs showing emotionally matching (M) and mismatching (MM) displays. In each run participants were shown 24 stimuli, of which 6 were emotionally matching and 18 were emotionally mismatching, for a total of 72 presentations. The emotionally matching displays combined visual and auditory signals that expressed the same emotion but were extracted from different original audiovisual displays. In contrast, the emotionally mismatching displays combined visual and auditory information that expressed different emotions and was extracted from different original audiovisual displays. In this way both types of emotionally matching and mismatching stimuli were presented as asynchronous visual and auditory signals, as illustrated in the temporal profiles at the bottom of Figure 1 (right).

### Data Acquisition and Image Analysis

T1 and T2* weighted scans were acquired using a 3T Tim Trio Siemens scanner. The functional scan consisted of three runs for each experiment (TR = 2000 ms; TE = 30 ms; 32 Slices; 3 mm^3^ isovoxel; 70×70 image resolution; 216 Volumes (Exp1), 192 Volumes (Exp2)). The anatomical scan of the whole brain structure was acquired using a 3D MP-RAGE T1-weighted sequence (192 slices; 1 mm^3^ isovoxel; Sagittal Slice; TR = 1900 ms; TE = 2.52; 256×256 image resolution).

The data were analysed by using Brain Voyager QX 1.9 (http://www.BrainVoyager.com). The functional data (DICOM format) were loaded and converted into Brain Voyager's internal FMR data format. A standard pipeline of pre-processing of the data was performed for each participant [17]. Slice scan time correction was performed using sinc interpolation based on information about the TR and the order of slice scanning. In addition, 3-D motion correction was performed to detect and correct for small head movements by spatial alignment of all the volumes of a participant to the first volume by rigid body transformations. Estimated translation and rotation parameters never exceeded 3 mm. Finally, the functional MR images were smoothed using a Gaussian filter (FWHM = 8 mm). The anatomical data (DICOM format) of each participant were loaded and converted into Brain Voyager's internal VMR data format [17]. The data were then aligned with the AC-PC (anterior commissure - posterior commissure plane) and transformed into Talairach standard space. To transform the functional data into Talairach space, the functional time series data of each participant was first co-registered with the participant's 3-D anatomical data, followed by the same transformations of 3-D anatomical data applied to the functional data. This step results in normalised 4-D volume time course (VTC) data. Normalisation was performed by combining a functional-anatomical affine transformation matrix, a rigid-body AC-PC transformation matrix, and an affine Talairach grid scaling step. The alignment of the functional and anatomical data was then optimised by manual adjustment to reduce as much as possible the geometrical distortions of the images. For each run of each participant's event related data, a protocol file was uploaded representing the onset and duration of the events for the different conditions. From the created protocols, the design matrices for Experiment 1 and Experiment 2 were defined automatically and each predictor was derived by convolution with a haemodynamic response function.

#### Whole-brain analysis

Statistical evaluation of group data was based on a second-level GLM random effects analysis. For the conjunction analysis performed in Experiment 1 the activations are reported at a threshold of *P*<0.01 (uncorrected), corrected using the cluster-size threshold of *P*<0.05 [17] based on a 3D extension of the randomisation procedure [18]. The threshold for the conjunction analysis was kept lower than for simple contrast analysis because conjunction analyses are very conservative strategies [7]. When performing simple contrasts between the stimulus conditions and baseline across the whole brain the resulting map was corrected for multiple comparisons by applying a Bonferroni correction of p<0.05. Peaks are specified as Talairach coordinates (x, y, z).

#### ROI-analysis

To test whether any of the areas obtained in Experiment 1 participated in the detection of emotional mismatch, we performed a ROIs analysis and contrasted the activity elicited in these areas by emotionally mismatching displays with that elicited by emotionally matching displays. To examine the time-course of BOLD activity associated with the different stimulus conditions we performed event-related averaging for the different experimental conditions. This resulted in a plot of percentage BOLD signal change relative to a baseline. The baseline was calculated by first finding the pre-period BOLD value at each trial and then averaging all these pre-period values over the entire time course.

## Results

### Experiment 1: Emotional classification in the scanner

**Figure 2:**
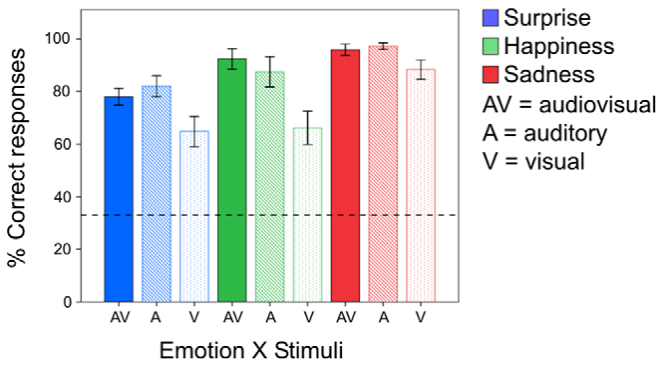
Percentages of correct emotional classification obtained in the scanner during Experiment 1. The percentages are shown for the three emotions – surprise (blue), happiness (green), and sadness (red) – and the three stimuli conditions – audiovisual (AV), auditory (A), and visual (V). The error bars represent the standard error of the mean and the dashed line the level of chance performance.

The first step was to make sure that participants were perceiving the intended emotional valence when viewing the displays during the scan. During the scan participants were asked to judge the perceived emotion while presented with audiovisual (AV), auditory–only (A), and visual–only (V) musical displays (Figure 1 A). The collected emotional judgements given by all participants were analysed by carrying out a 3 (experimental runs)×3 (emotions: sadness, happiness, and surprise)×3 (stimuli: audiovisual, auditory–only, and visual–only) repeated measures ANOVA. Figure 2 shows the averaged classification data for emotion x stimuli collapsed across the different runs. Participants correctly perceived the intended emotion far above the level of chance (33%) for all the nine stimuli conditions (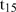
≥5.207, p<0.001, two-tailed). The ANOVA showed a significant effect of runs (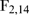 = 17.062, p<0.001) and of emotion (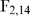 = 22.211, p<0.001). Repeated contrast measures (corrected for multiple comparisons) showed that while there was no significant difference between the correct percentage of emotional judgements given by subjects at run 1 and 2 (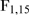 = 4.739, p = 0.092), there was a significant increase in correct responses when going from run 2 to 3 (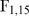 = 19.286, p = 0.001). This indicates a certain degree of learning during the experiment, which supports the idea of participants being unfamiliar with the excerpts of music improvisation used in our study. Also, repeated contrast measures showed that there was no significant difference between the number of correct emotional classifications of happiness and surprise (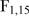 = 3.889, p = 0.134), while there was a significant difference between those of happiness and sadness (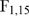 = 8.515, p = 0.022). Hence, the effect of the within-subjects variable ‘emotions’ on the correct classification rate was due to the higher number of correct responses for the sad musical displays (Figure 2) than for either the happy or surprise displays. The within-subjects variable ‘stimuli’ was also significant (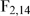 = 26.429, p<0.001) and simple contrast measures (corrected for multiple comparisons) confirmed the observation (Figure 2) that this effect was driven by the lower number of correct classifications received by the visual–only displays with respect to the audiovisual (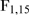 = 19.539, p<0.001), in that no difference in correct classification rate was found between the auditory–only and audiovisual stimuli (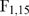 = 0.005, p = 0.943).

These behavioural results replicate our previous findings [4] and support the idea that in musical stimuli the auditory information is dominant over the visual, a result that is complementary to that found for face–voice emotional classification tasks [19], in which the visual information may dominate the auditory. Finally, no significant effect of interaction between ‘emotion’ and ‘stimuli’ (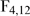 = 2.074, p = 0.148), between ‘run’ and ‘stimuli’ (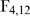 = 1.385, p = 0.297), or between ‘run’ ‘emotion’ and ‘stimuli’ (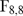 = 2.231, p = 0.139) was found. However, we did find a significant interaction between ‘run’ and ‘emotion’ (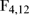 = 7.335, p = 0.003), and contrast measures showed that this effect was due to the decrease in difference between the correct responses given to surprise and happy displays when going from run 1 to run 2 (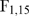 = 11.733, p = 0.004). No difference between these two emotions was found when going from run 2 to run 3 (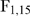 = 2.718, p = 0.120). Nor was any significant difference found between the percentage of correct responses given to the happy and sad displays when going from run 1 to 2 (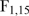 = 0.849, p = 0.371), or from run 2 to 3 (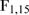 = 0.944, p = 0.347).

### Experiment 2: Emotional classification in the scanner

In the second experiment participants were asked to judge, once again, the perceived emotion while being presented with emotionally mismatching and matching displays. The emotionally mismatching displays were obtained by combining the visual information of each display used in the first experiment (e.g. musician's movements from a surprise display) with the auditory information from each one of the other displays representing a different emotion (e.g. sound from a happiness display: right hand side of Figure 1B). In contrast, the emotionally matching displays were obtained by combining the visual information of each display used in the first experiment (e.g. musician's movements from a surprise display) with the auditory information from each one of the other displays representing the same emotion (e.g. sound from another surprise display: left hand side of Figure 1B). In this way, not only was the same visual and auditory information presented in both display conditions, but the auditory and visual temporal correspondence (the natural synchronisation between the musician's movements and the resulting sound) was also eliminated for both emotionally matching and mismatching displays. This was necessary to avoid the brain activation being elicited by differences in audiovisual temporal correspondence between the emotionally matching and mismatching displays [20].

Because there is no unique way to classify the perceived emotion of mismatching audiovisual stimuli, we scored a participant response as correct when it was identical to the auditory emotional content. By running a 3 (experimental runs)×2 (stimuli: emotionally mismatching and matching) repeated measures ANOVA we compared, for the three separate runs, the correct auditory judgements given when the visual information matched emotionally with those given when the visual information mismatched. The results showed a tendency towards choosing the correct emotion more often when the visual emotional information matched the auditory (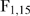 = 4.290, p = 0.056), but no significant effect of run (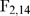 = 0.906, p = 0.427) and no interaction between run and stimuli (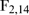 = 0.697, p = 0.515).

### fMRI Results

#### Experiment 1: Whole brain analysis

The brain activation elicited by auditory (A), visual (V) and audiovisual (AV) stimuli was compared to baseline in order to detect the areas that would respond to these kinds of stimuli. Hence, we carried out three contrasts (A>0, V>0, and AV>0) and corrected the results for multiple comparisons (Bonferroni: p<0.05). Most of the obtained regions showed activation during all three stimulus conditions, e.g. left thalamus, left precentral gyrus, left inferior parietal lobule. However, a few regions were detected specifically for only one of the three conditions, e.g. the left putamen for auditory only information, the inferior occipital gyrus for visual only information, and the right thalamus for audiovisual information. The anatomical location and details of the activated foci are listed in Table 1. The simple contrast AV>V returned auditory and multisensory areas while the contrast AV>A returned visual and multisensory areas. Figure S1 shows the auditory and visual areas returned by the contrasts and the respective time courses for the audiovisual, auditory and visual conditions.

**Table 1:** Experiment 1 clusters of activation for audio >0, visual >0 and audiovisual >0.

Anatomical region	Hemisphere	Talairach	Number of voxels	Effect size^a^	BA^b^
***Audio>0***				*t(15) P*<0.0001	
Superior temporal gyrus	Right	50, −6, 4	5745	9.442	22
Insula	Right	33, 20, 11	827	9.128	13
Lingual gyrus	Right	5, −77, −7	4501	9.116	18
Cuneus	Right	7, −96, 12	16	8.419	18
Thalamus	Left	−11, −17, 3	188	8.568	
Putamen	Left	−17, 4, 11	20	8.343	
Cerebellum (Declive)	Left	−25, −68, −17	95	8.632	
Precuneus	Left	−29, −62, 38	127	8.635	19
Insula	Left	−33, 17, 12	865	9.045	13
Precentral gyrus	Left	−31, −19, 54	203	8.632	4
Inferior Parietal lobule	Left	−36, −39, 49	1776	8.928	40
Insula	Left	−42, 4, 1	860	8.810	13
***Visual>0***				*t(15) P*<0.0001	
Inferior Occipital gyrus	Right	39, −69, −7	2478	9.211	19
Fusiform gyrus	Right	35, −41, −16	1540	9.147	20
Insula	Right	33, 22, 10	154	8.509	13
Cuneus	Right	18, −93, 5	3230	9.697	17
Cerebellum	Right	4, −79, −15	244	8.702	
Thalamus	Left	−11, −14, 6	27	8.518	
Middle occipital gyrus	Left	−22, −90, 5	1619	9.016	18
Precuneus	Left	−23, −77, 28	19	8.444	31
Cerebellum (Declive)	Left	−26, −67, −18	68	8.503	
Fusiform gyrus	Left	−40, −72, −10	1841	8.938	19
Inferior Parietal lobule	Left	−37, −37, 48	4070	9.131	40
Precentral gyrus	Left	−31, −19, 54	392	8.848	4
Precuneus	Left	−29, −62, 38	148	8.622	19
Precentral gyrus	Left	−55, 2, 36	26	8.402	6
***Audiovisual>0***				*t(15) P*<0.0001	
Superior temporal gyrus	Right	52, −21, 6	2520	8.823	41
Cerebellum (Declive)	Right	16, −76, −15	2589	9.264	
Cerebellum (Culmen)	Right	32, −44, −16	627	8.936	
Cuneus	Right	18, −92, 4	1605	8.979	17
Thalamus	Right	12, −12, 2	29	8.465	
Thalamus	Left	−12, −17, 4	368	8.670	
Middle occipital gyrus	Left	−19, −96, 6	449	8.665	18
Middle occipital gyrus	Left	−24, −83, 4	203	8.624	18
Precuneus	Left	−30, −62, 38	109	8.598	19
Precentral gyrus	Left	−31, −17, 54	165	8.561	4
Fusiform gyrus	Left	−40, −74, −13	916	9.017	19
Inferior Parietal lobule	Left	−37, −41, 46	1548	9.004	40
Insula	Left	−35, −3, 4	488	9.557	13
Postcentral gyrus	Left	−37, −30, 53	219	8.473	3

a Effect size = average *F* value for all voxels in the ROI.

b Brodmann area.

#### Experiment 1: Conjunction analysis and interaction analysis

We performed a conjunction analysis – (AV>A)∩(AV>V) – to find the brain regions that responded with higher activation to the audiovisual displays. We report the conjunction analysis results here instead of the interaction analysis – AV>A+V – because although the latter method detected positive interactions, these resulted from a deactivation of the haemodynamic response to all three conditions (see Figure S2 for an example and Ethofer et al, [7] for a review). Also we used conjunction analysis since this method has previously been used to detect brain regions that responded with higher activation to emotional audiovisual speech stimuli than either visual or auditory stimuli [7], [8]. Thus running a similar analysis allows us to easily compare our findings with music stimuli to those using speech stimuli. The conjunction analysis showed greater activation in the right thalamus (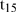 = 2.95, p<0.01, two-tailed; 11, −25, 1 (x, y, z); 265 voxels) and right culmen (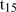 = 3.17, p<0.01, two-tailed; 2, −40, 4 (x, y, z); 334 voxels). This result is in line with the findings collected by carrying out the whole brain analysis.

**Figure 3:**
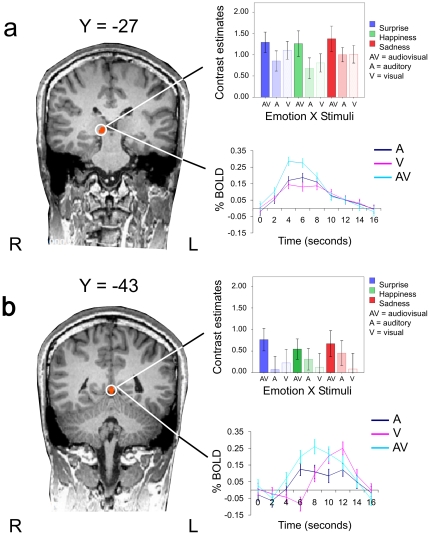
The coronal slices show the right thalamus and right culmen activation at two y Talairach co-ordinates. Activation assessed in Experiment 1 by contrasting the brain response for the audiovisual displays with that for the auditory– and visual–only displays: (AV>A)∩(AV>V). (**a**) The averaged contrast estimates for the audiovisual, auditory and visual conditions are reported separately for the three emotional categories (sadness, happiness, and surprise). The audiovisual emotional conditions (thalamus: mean = 1. 309±0.248; culmen: 0.661±0.242) activated these areas more than either the auditory (thalamus: mean = 0.844±0.155; culmen: 0.279±0.248) or visual conditions (thalamus: mean = 0.974±0.168; culmen: 0.143±0.316), similarly for all the portrayed emotions. (**b**) Event-related bold signals are shown for auditory (red), visual (green) and audiovisual (blue). The error bars represent the standard error of the mean. L = left hemisphere; R = right hemisphere.

The observed integration effect in the right thalamus (Figure 3a) and right culmen (Figure 3b) was further examined by carrying out a 3 (emotion: sadness, happiness, surprise)×3 (stimuli: audiovisual, auditory, visual) ANOVA for repeated measures on the derived contrast estimates (averaged beta weights). The within-subjects factor ‘emotion’ did not reach significance for either region (Thalamus: 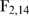 = 1.101, p = 0.360; Culmen: 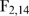 = 0.455, p = 0.643), indicating that the displays representing the three different emotions elicited a similar response in these areas. The within-subjects factor ‘stimuli’ was on the contrary significant for both regions (Thalamus: 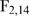 = 4.729, p = 0.027; Culmen: 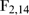 = 5.265, p = 0.020), while no significant interaction between ‘emotion’ and ‘stimuli’ was found (Thalamus: 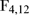 = 0.229, p = 0.917; Culmen: 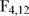 = 0.836, p = 0.528). These results indicated that the audiovisual condition activated these areas more than either the auditory or visual condition similarly for all the portrayed emotions (Figure 3). However, when performing a ROI analysis on the right thalamus and culmen to examine whether these areas would respond differently to emotional mismatching and matching displays presented during Experiment 2, no significant difference was found (Thalamus: 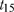 = 1.308, p = 0.191, two-tailed; Culmen: 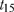 = 0.438, p = 0.661, two-tailed). Furthermore, when running a 3 (stimuli: audiovisual, visual and auditory)×3 (experimental runs) repeated measures ANOVA on these two ROIs, no significant effect of runs (Thalamus: 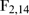 = 1.075, p = 0.368; Culmen: 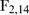 = 2.150, p = 0.153) or interaction between runs and stimuli (Thalamus: 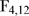 = 1.101, p = 0.400; Culmen: 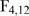 = 0.330, p = 0.852) was found. A significant effect of stimuli was instead found for both the thalamus (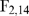 = 7.930, p = 0.005) and the culmen (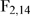 = 20.714, p<0.001).

#### Experiment 2: ROI analysis

**Figure 4:**
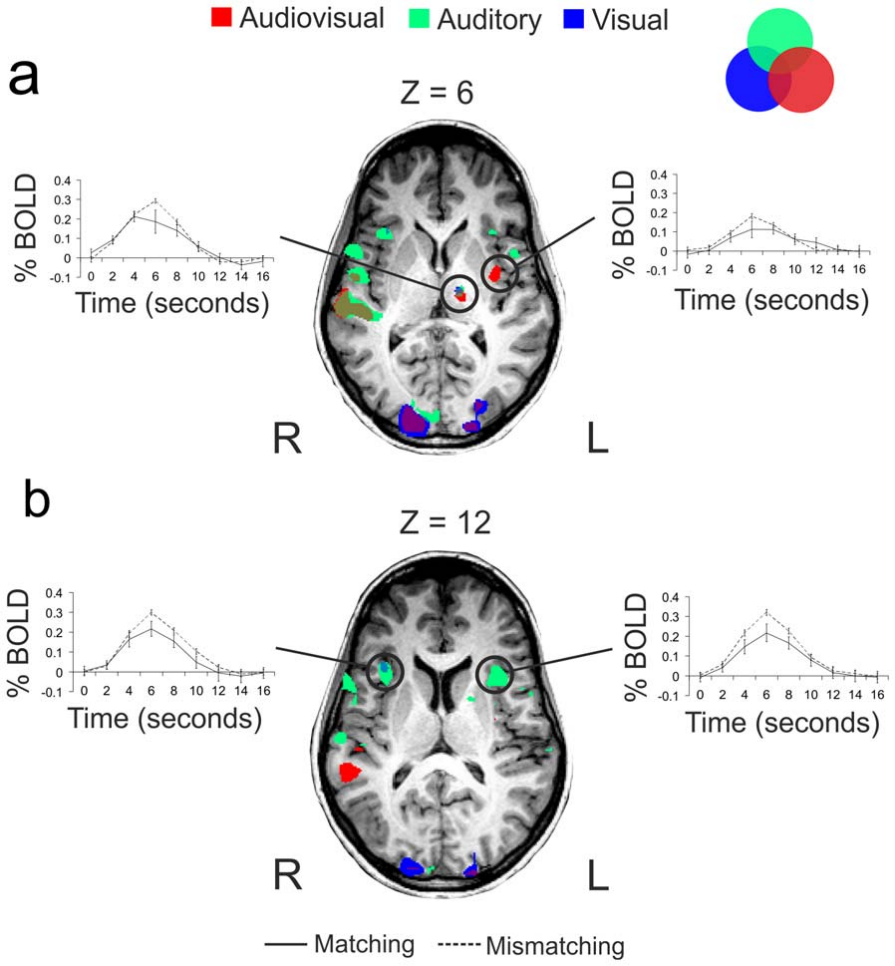
The axial slices show the left thalamus and bilateral insula activation at two z Talairach co-ordinates. Activations were assessed by contrasting the brain response for the audiovisual >0 (red), auditory >0 (green), and visual >0 (blue) in Experiment 1. The event-related bold signals are shown for the regions of interest that responded with greater activation to emotionally mismatching than matching stimuli as presented during Experiment 2. The mismatching and matching conditions are represented as dashed and solid line respectively and are presented for the larger cluster of activation when the conditions from Experiment 1 overlap (i.e. when the same area activated for A and V, A and AV, V and AV, and A, V and AV). **a**) Left thalamus and left posterior insula; **b**) bilateral anterior insula. The error bars represent the standard error of the mean. L = left hemisphere; R = right hemisphere.

The areas of brain activation obtained in Experiment 1 by contrasting emotional auditory (A), visual (V) and audiovisual (AV) stimuli with the baseline were selected as regions of interest (ROIs) for further analysis. We contrasted the activity elicited by emotionally mismatching displays (for which the sight and sound of musical actions mismatched in emotional content) with that elicited by emotionally matching displays (for which the sight and sound of musical actions matched in emotional content). We contrasted the activation elicited by all the emotional mismatching displays with the activation elicited by all emotional matching displays, instead of running this contrast separately for the three emotional categories (sadness, happiness and surprise). We did this because we wanted to make sure that any visual and auditory information was represented in the two groups of displays (emotionally matching and mismatching), thus excluding the possibility that any found difference in activation between these conditions was driven by differences in stimulus characteristics. The results indicated that only the bilateral insula, the left putamen and the left thalamus showed greater activation for mismatching than matching emotional displays (Figure 4), while no region of interest showed greater activation for the matching than the mismatching. The details of the ROI analysis for these regions are reported in Table 2.

**Table 2:** Regions of interest obtained from A>0, V>0 and AV>0 in Experiment 1 showing different activation for the emotionally matching and mismatching audiovisual displays.

Anatomical region	Hemisphere	Mismatching>Matching	
		*t(15)*	*P*
***Audio>0***			
Insula	Right	3.662	0.000252
Thalamus	Left	2.934	0.003354
Putamen	Left	2.582	0.009827
Insula	Left	4.617	0.000004
***Visual>0***			
Insula	Right	3.450	0.000564
Thalamus	Left	4.405	0.000011
***Audiovisual>0***			
Thalamus	Left	3.039	0.002381
Insula	Left	2.163	0.030581

## Discussion

The behavioural results show that the musical clips were capable of eliciting the intended emotions of sadness, surprise, and happiness. When the brain activity elicited by the presentation of audiovisual, visual and auditory displays was contrasted with the baseline, several regions were activated, including multisensory, auditory and visual areas (e.g. superior temporal gyrus, cuneus, precentral gyrus). However, only two of these regions, the right thalamus and right culmen, responded with greater activity to audiovisual emotional displays, and only four of these regions, the bilateral insula, left thalamus, and left putamen, discriminated between emotionally matching and mismatching audiovisual displays.

### Fusion of emotional signals from music

Experiment 1 shows that the right thalamus and culmen activate more for audiovisual emotional music displays than either auditory only or visual only displays. Blood and Zatorre [1] found the rCBF in the right thalamus to increase in response to an increase of chills intensity from music, and Ethofer et al. [7] and Kreifelts et al. [8] indicated that the right thalamus is involved in the audiovisual integration of emotional signals from speech. Our findings add relevant information about the function of this area by indicating how the right thalamus may subtend the audiovisual integration of emotional signals from music as well as from speech. That processing of multisensory music and speech information involves common brain networks was suggested by the similar enhancement music practice has been found to have on the early encoding of both kinds of information [21]. Our findings support the idea that to some extent the processing of speech and music multisensory information overlaps, and they extend this possibility to the processing of emotional information. The right thalamus, however, did not discriminate between emotionally mismatching and matching stimuli for which the temporal covariation between the sensory information was eliminated. The sensitivity of the right auditory thalamus to audiovisual simultaneity is now well known; for example, Komura et al. [22] showed that a visual stimulus needed to occur in synchrony with the sound for the response of the right auditory thalamus to be modulated. Our findings thus add further support to this view by showing that the right thalamus does not respond with different activity to audiovisual stimuli that do not match temporally, even when they do match in emotional content.

It is interesting also to note that the greater activation we found in the right thalamus for the audiovisual displays than for either the auditory or visual displays does not reflect facilitation for the audiovisual condition at the behavioural level. Indeed, the auditory-only displays were perceived as eliciting the intended emotion as much as the audiovisual displays [4], although the contrast parameters for the auditory-only condition in the right thalamus showed the lowest level of activation. This is an intriguing result which suggests that achieving a multisensory facilitation effect in the classification of emotional signals [8] is not necessary in order for the right thalamus to respond more actively to the emotional information when presented bimodally. On the other hand, the lack of a facilitation effect in classification for the audiovisual condition may be the reason why we did not find greater activation for these stimuli in the posterior superior temporal gyrus (pSTG). This region has been found to activate consistently when contrasting audiovisual speech stimuli with unimodal stimuli representing the same emotional content [7], [8]. Furthermore, previous inspection of contrast estimates showed that the behavioural facilitation for the audiovisual condition of seven different emotional categories was better represented by the pattern of activation in the bilateral pSTG than in the right thalamus [8].

In addition to the right thalamus, the right culmen was also found to have greater activation for the audiovisual displays than either the auditory or visual displays. The culmen is part of the cerebellum, and since the right cerebellum has been previously found to activate more for audiovisual conditions than either auditory or visual in a face–voice matching task [23], and has been found to increase its response with increasing chills intensity from music [1], our results support the involvement of this area in the audiovisual fusion of moving visual and auditory musical stimuli. Also, the same area in the left hemisphere was found to activate more when participants were presented with pleasant odorant than when they were resting (−2, −41, −3 (x, y, z)) in a study by Wicker et al. [24], demonstrating the participation of this area in more general emotional processes.

### Correspondence of emotional signals from music

The ROI analysis performed on the areas detected in Experiment 1 indicated that the bilateral insula, the left thalamus and the left putamen had a greater response to mismatching than matching emotional displays. The activation of the putamen and left thalamus has recently been found to correlate with the tempo of expressive music performances, suggesting that these two motor-related areas may be involved in emotional response to music guided by temporal expectancy [25]. In the present study, the displays that mismatched in audiovisual temporal and emotional features activated these regions significantly more than those that mismatched in only temporal features, thus supporting and extending these recent observations [25].

The insula is known for being involved in a wide range of processes [9]; nonetheless this region is a constant when it comes to investigating the processes underlining music-evoked emotions [1], [9]–[13], [26]. In our study, three different regions of the insular cortex responded with greater activation to emotionally mismatching than emotionally matching displays, the left and right anterior insular cortex (AIC) and a more posterior portion of the left insula. The AIC is not only crucial for processing emotions from music [1], [9]–[13], as indicated by the consistent association between damage to this structure and altered emotional responses to music [10], [27], but it is also crucial for processing emotions from social situations [9], [28]–[29]. Indeed, whereas the right AIC is assumed to be generally activated by stimuli that arouse the body, the left AIC is activated by affiliative emotional feelings [9]. Interestingly, in our study, the right AIC was activated by both the sight and sound of emotional stimuli, while the left AIC was activated only by the sound of the emotional music displays. This functional asymmetry suggests that while the sound might induce both individual and affiliative feelings [9], seeing the musician's movements might have a more specific effect on the observer's body response.

Finally, another more posterior region of the left insula was found to activate more for emotionally mismatching than matching displays. The activity in this region was detected specifically when contrasting the audiovisual condition against the baseline in Experiment 1, and its peak coordinates are very similar to those found by Buccino et al. [30] for the execution of non-related actions to observed hand movements on a guitar, by Baumgartner et al. [13] for combined pictures and music against pictures only, and by Naghavi et al. [31] for conceptually related sounds and pictures. Hence, this region of the posterior left insula appears to have specific somatosensory and multisensory functions [32] that together with its anatomical position make it the perfect node of transition for the information coming from the left thalamus and putamen and going to the left AIC [9].

The connections between the insular cortex and the thalamus have been described in detail by Mufson and Mesulam [32], who showed that the thalamic nuclei of rhesus monkeys receive projections from both the posterior and anterior insular cortex. In the present study the medial division of the ventral lateral nucleus of the thalamus and the anterior insula were found to both activate bilaterally for our musical stimuli, suggesting an interaction between these regions. Since the two nuclei of the thalamus appear to have different multisensory and limbic functions, this might reflect different functions of the right and left anterior insula. This possibility is supported by the asymmetric response of the anterior insula to the visual stimuli. A functional differentiation would support the idea of Miller and D'Esposito [33] that there are separate networks of audiovisual integration. Indeed, whereas the right thalamus and right anterior insula may be part of an audiovisual fusion network, the left thalamus and left anterior insula may be part of an audiovisual correspondence network [33]. This differentiation between a mechanism subtending audiovisual integration and one subtending audiovisual correspondence is also supported by the finding that a greater activity for mismatching emotional displays than matching was found in ROIs that activated specifically for unimodal information in Experiment 1. That is, for example, the left anterior insula was detected in Experiment 1 when contrasting A>0, nevertheless this area responded with more activation to emotionally mismatching than matching audiovisual displays. Based on our results, however, we cannot draw conclusions on the way the thalamus and insula interact during the integration and detection of correspondence between the sensory signals, though connectivity studies could shed light on this point.

### Conclusion

Our findings show that the emotional content of musicians' body movements modulates the brain activity elicited by the emotional content of the musical sound. This result might have a social origin, as suggested by a recent study of entrainment in children [34]. This study showed that young children find it difficult to entrain to a purely auditory rhythmic stimulus, or to a visible drum-beating robot. They nonetheless entrain when they can interact with another human adult. In a similar way, the movements of musicians can facilitate music enjoyment and emotional participation [3], [4]. Hence the role of these areas in detecting emotional correspondence between music and others' body movements may reflect the adaptive function of music in influencing our emotions in social contexts. More conclusive results on this point might be achieved in future studies by introducing a controlled ‘non-social’ and ‘non-emotional’ condition in order to test whether the insula, left thalamus, and left putamen are specifically involved in the detection of audiovisual correspondence of socially relevant stimuli.

## Supporting Information

Figure S1The axial slices show activation bilaterally in auditory (right: 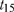 = 6.86, *p* = 0.00001, two-tailed; 53, −14, 5 (x, y, z); 11336 voxels; left: 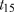 = 6.23, *p* = 0.00002, two-tailed; −48, −20, 7 (x, y, z); 6781 voxels) and visual areas (right: 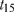 = 6.59, *p* = 0.00002, two-tailed; 32, −76, −8 (x, y, z); 24445 voxels; left: 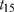 = 6.41, *p* = 0.00002, two-tailed; −31, −82, −9 (x, y, z); 14862 voxels) at two peak z Talairach co-ordinates and at a false discovery rate (FDR) threshold <.005. The event-related responses to audiovisual (light blue), auditory (dark blue) and visual (magenta) stimuli are presented at the bottom of the figure. The error bars represent the standard error of the mean. L = left hemisphere; R = right hemisphere.(TIF)

Figure S2 Example of one region detected by using interaction analysis AV>A+V. The region (left parahippocampal gyrus: x = −25; y = −18; z = −14) is presented on the left and the relative time courses on the right. L = left hemisphere; R = right hemisphere.
(TIF)
