# Supplementary figures and images for: The Music of Your Emotions: Neural Substrates Involved in Detection of Emotional Correspondence between Auditory and Visual Music Actions
Source: PLoS One. 2011 Apr 29;6(4):e19165. doi: 10.1371/journal.pone.0019165 (PMC3084768; doi:10.1371/journal.pone.0019165)

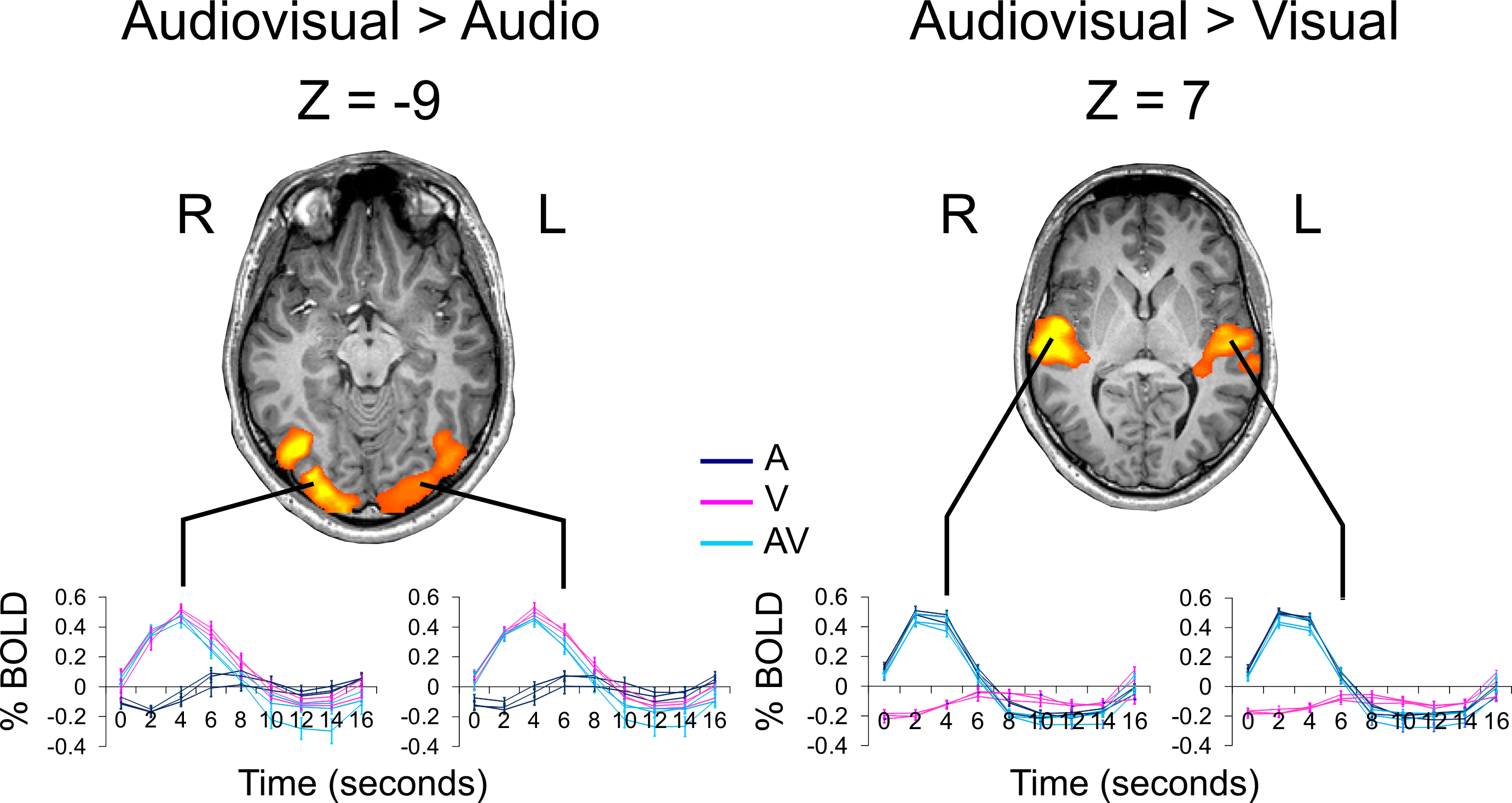

Supplement: Figure S1 — The axial slices show activation bilaterally in auditory (right: = 6.86, p = 0.00001, two-tailed; 53, −14, 5 (x, y, z); 11336 voxels; left: = 6.23, p = 0.00002, two-tailed; −48, −20, 7 (x, y, z); 6781 voxels) and visual areas (right: = 6.59, p = 0.00002, two-tailed; 32, −76, −8 (x, y, z); 24445 voxels; left: = 6.41, p = 0.00002, two-tailed; −31, −82, −9 (x, y, z); 14862 voxels) at two peak z Talairach co-ordinates and at a false discovery rate (FDR) threshold <.005. The event-related responses to audiovisual (light blue), auditory (dark blue) and visual (magenta) stimuli are presented at the bottom of the figure. The error bars represent the standard error of the mean. L = left hemisphere; R = right hemisphere. (TIF) [file pone.0019165.s001.tif]

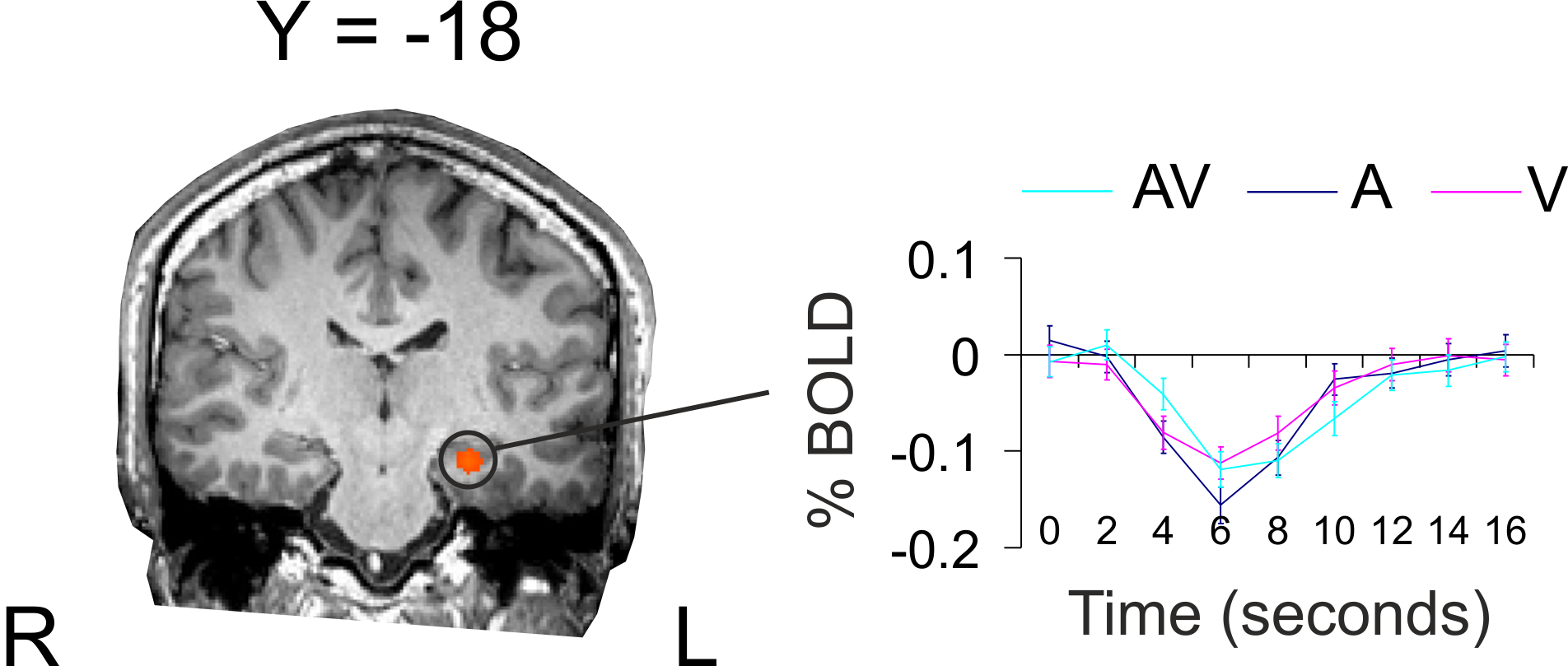

Supplement: Figure S2 — Example of one region detected by using interaction analysis AV>A+V. The region (left parahippocampal gyrus: x = −25; y = −18; z = −14) is presented on the left and the relative time courses on the right. L = left hemisphere; R = right hemisphere. (TIF) [file pone.0019165.s002.tif]
